# Risk Factors for Intestinal Barrier Impairment in Patients With Essential Hypertension

**DOI:** 10.3389/fmed.2020.543698

**Published:** 2021-01-27

**Authors:** Cao Li, Ping Xiao, Da Lin, Hao-Jie Zhong, Ran Zhang, Zhi-gang Zhao, Xing-Xiang He

**Affiliations:** ^1^Department of Pharmacy, Beijing Tiantan Hospital, Capital Medical University, Beijing, China; ^2^Department of Gastroenterology, The First Affiliated Hospital of Guangdong Pharmaceutical University, Guangzhou, China; ^3^Graduate School, Guangdong Medical University, Zhanjiang, China

**Keywords:** hypertension, risk factor, intestinal barrier, gut microbiota, prediction

## Abstract

**Background:** Previous studies have indicated an association between hypertension and intestinal barrier dysfunction in mice models. The present study aims to investigate the association between hypertension and intestinal barrier impairment in humans and identify the novel potential risk factors for hypertension.

**Methods:** Medical data from consecutive inpatients were retrospectively pooled from patient records. We compared intestinal barrier serum markers [diamine oxidase (DAO), lipopolysaccharide (LPS), and _D_-lactate] between those patients with and without hypertension. Moreover, the associations between intestinal barrier markers and cardiovascular risk, hypertension history, blood pressure control, hypertensive complications, and antihypertensive medication history were also analyzed.

**Results:** Overall, 106 hypertensive and 251 normotensive subjects were included. Patients with hypertension had a higher level of DAO (28.30 vs. 18.73%, *P* = 0.044) and LPS (22.64 vs. 11.16%, *P* = 0.005). In hypertensive patients, multivariate logistic regression analyses showed that long hypertension history (≥20 years), poor control of diastolic blood pressure, cardiac and renal complications, and use of multiple antihypertensive medications were risk factors for elevated DAO, while the use of multiple antihypertensive medications was a risk factor for elevated _D_-lactate (*P* < 0.05).

**Conclusions:** Hypertension is associated with impairment of intestinal barrier, especially in patients with long duration, poor blood pressure control, cardiac and renal complications, and use of multiple antihypertensive medications. The current study indicates that intestinal barrier dysfunction might be a potential predictor of hypertension.

## Background

Hypertension is a significant public health issue worldwide, which is the strongest modifiable factor related to cardiovascular disease and strokes, causing high morbidity and mortality (1, 2). The global prevalence of hypertension was about 1.13 billion in 2015, and it is estimated to rise to 1.56 billion by 2025 (3).

The association between the gut and cardiovascular disease has begun to draw our attention in recent years (4, 5). The concepts of the “gut–heart” and “gut–kidney” axes link the gut to blood pressure (BP) (6, 7). As the largest mucosal surface in the body, the intestinal barrier maintains host homeostasis by providing a barrier against luminal bacteria, dietary antigens, and toxins (5). Intestinal barrier impairment may trigger or exacerbate many diseases including infections, metabolic diseases, and cancer (8–10). Additionally, multiple diseases can also cause intestinal barrier alterations. The spontaneously hypertensive rat (SHR) model shows obvious alterations in intestinal pathology, including decreased goblet cells and villi length and increased gut permeability and inflammation, suggesting that hypertension led to intestinal barrier impairment (11, 12).

Endoscopy is common used to diagnose intestinal barrier impairment, only severe injuries such as erosion, ulcers, and bleeding can be detected. Early-stage damage, such as small intestinal epithelium injury, endotoxin translocation, and increased intestinal permeability, is difficult to detect. Serum diamine oxidase (DAO) (13, 14), lipopolysaccharide (LPS) (15, 16), and _D_-lactate (14, 17, 18) are convenient and well-accepted biomarkers for intestinal barrier function.

Currently, the correlation between hypertension and intestinal barrier impairment is far from known. The present study aims to investigate the role of typical serum markers of intestinal barrier function in hypertensive patients and to find novel potential risk factors for predicting and treating hypertension.

## Methods

### Study Population

In this retrospective study, we reviewed the medical data of all inpatients in the Department of Gastroenterology of the First Affiliated Hospital of Guangdong Pharmaceutical University treated from January to December 2017. The exclusion criteria were as follows: (i) carcinoma; (ii) severe heart, lung, liver, or kidney disease, except hypertensive cardiac or renal complications; (iii) non-steroidal anti-inflammatory drug use in the previous month; (iv) intestinal diseases such as ulcerative colitis, Crohn's disease, or intestinal obstruction; (v) history of gastrointestinal surgery; (vi) bacterial infection, except *Helicobacter pylori*; and (vii) secondary hypertension caused by renal parenchymal, adrenal, renovascular, or thyroid disease. Patients hospitalized more than once during the study period were recorded only once. A total of 1372 patients were detected with the three serum biomarkers in the department of gastroenterology in 2017, and a final 357 subjects were included in the present study.

This study was performed in accordance with the Declaration of Helsinki and with the approval of the Ethical Committee of the First Affiliated Hospital of Guangdong Pharmaceutical University. Given that this was a retrospective study, informed consent from the research subjects was waived.

### Data Collection

We collected data from patient records on demographics (age and gender), height, and weight [to calculate the body mass index (BMI)]. We also collected data on hypertension-related factors including hypertension status, cardiovascular risk, duration, BP control, complications, and antihypertensive medication use. Diagnosis of hypertension was based on the classic criteria: systolic BP (SBP) ≥140 mmHg and/or diastolic BP (DBP) ≥90 mmHg (19). Subjects who did not meet these criteria were categorized as normotensives. Cardiovascular risk assessment was based on demographic characteristics, laboratory parameters, hypertension-mediated organ damage, and comorbidities. Good BP control was defined as SBP ≤130 mmHg and DBP ≤80 mmHg. Hypertensive complications comprised cardiac complications (left ventricular hypertrophy, severe arrhythmias, and heart failure), vascular complications (atherosclerosis, arteriosclerotic stenosis, and aortic aneurysm) (20), renal complications (proteinuria, increased serum creatinine, and increased blood urea nitrogen), and cerebral complications (hemorrhagic and ischemic strokes).

We also collected data on the history of smoking, alcohol consumption, history of liver diseases (fatty liver disease or any kind of hepatitis), history of diabetes, cholesterol level, *H. pylori* status, and three intestinal barrier markers. Smokers were defined as patients who had smoked at any stage in their life. Alcohol consumption was defined as drinking >140 g of alcohol per week. Hypercholesterolemia was defined as serum cholesterol >5.2 mmol/L (21). *H. pylori* infection was diagnosed based on gastric mucosa pathobiology, a ^13^C-urea breath test, and a rapid urease test. Elevated DAO, LPS, and _D_-lactate were defined as ≥15, ≥20, and ≥10 U/L, respectively.

### Statistical Analysis

IBM SPSS software version 22 (IBM Corp., Armonk, NY, USA) was used to perform statistical analyses. Categorical variables are presented as frequencies and proportions. Non-normally distributed continuous variables are presented as medians and interquartile ranges. The statistical significance levels of differences between the hypertensive and normotensive subjects were tested using Chi-square or Fisher's exact tests for categorical variables and Mann–Whitney *U*-tests for medians. To identify risk factors for elevated intestinal barrier markers, we performed logistic regression analyses with a backward stepwise approach. Odds ratios (ORs) and 95% confidence intervals (CIs) were estimated. *P* < 0.05 (two-sided) was considered statistically significant.

## Results

### Demographics and Clinical Characteristics

In total, 357 patients (251 without hypertension and 106 with hypertension) were included, but specific subsets were used in different analyses (detailed below) due to patients having missing data for some variables. The demographics and clinical characteristics of the included patients were shown in Table 1.

**Table 1:** Patient demographics and clinical characteristics.

	**Hypertension**	**No hypertension**	***P*-value**
	**(*n* = 106)**	**(*n* = 251)**	
Age (years)	63.0 (57.0–72.0)	56.0 (46.0–63.0)	<0.001
Male gender	61 (57.55)	131 (52.19)	0.354
Body mass index (kg/m^2^)	23.73 (21.78–25.98)	22.23(20.00–24.91)	0.001
Disease duration (years)	(*n* = 82)		
<20	69 (84.15)		
≥20	13 (15.85)		
Diabetes status	24 (22.64)	17 (6.77)	<0.001
Liver disease	48 (45.28)	75 (29.88)	0.005
Alcohol consumption	12 (11.32)	26 (10.36)	0.788
History of smoking	23 (21.70)	46 (18.33)	0.461
Hypercholesterolemia	29 (31.87)	72 (33.64)	0.763
	(*n* = 91)	(*n* = 214)	
*Helicobacter pylori* infection	24 (22.64)	64 (25.50)	0.567

*Data are presented as medians (interquartile ranges) or n (%)*.

### Elevated DAO and LPS but Not _D_-lactate in Hypertensive Patients

**Figure 1:**
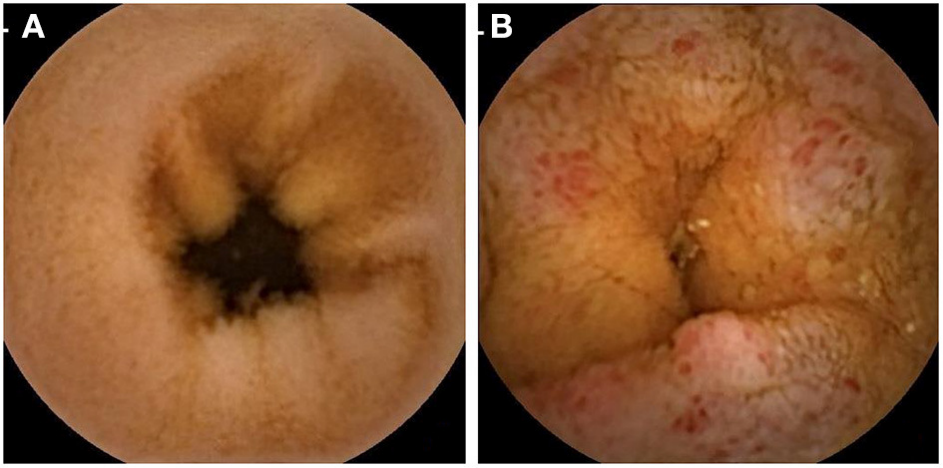
Representative capsule endoscopic images. **(A)** Normal small intestine of normotensive participants; **(B)** Mucosal edema (enlarge villi) and moderate to severe mucosal hyperemia (area of reddish villi) were observed in the small intestine of patients with hypertension.

To detect the association between intestinal barrier impairment and hypertension, two groups of hypertension and no hypertension were analyzed with DAO, LPS, and _D_-lactate. Table 2 shows that patients with hypertension had a significantly higher prevalence of elevated DAO (28.30 vs. 18.73%, *P* = 0.044) and elevated LPS (22.64 vs. 11.16%, *P* = 0.005) but not _D_-lactate (71.71 vs. 68.13%, *P* = 0.504). After adjustment for gender, age, BMI, smoking, alcohol consumption, liver disease, diabetes, and *H. pylori* infection, the logistic regression analysis showed that hypertension was a risk factor for elevated DAO (OR = 1.71, 95% CI: 1.01–2.91, *P* = 0.046) and elevated LPS (OR = 2.64, 95% CI: 1.42–4.92, *P* = 0.002). In addition, representative images of capsule endoscopy showed that edema, hyperemia, and mucosal lesions were frequently detected in the small intestine of patients with hypertension, whereas they were rarely observed in those participants without hypertension (Figure 1).

**Table 2:** Intestinal barrier markers in patients with or without hypertension.

	**Hypertension**	**No hypertension**	***P*-value**
	**(*n* = 106)**	**(*n* = 251)**	
Elevated DAO	30 (28.30)	47 (18.73)	0.044
Elevated LPS	24 (22.64)	28 (11.16)	0.005
Elevated _D_-lactate	76 (71.70)	171 (68.13)	0.504

*Data are presented as n (%). DAO, diamine oxidase; LPS, lipopolysaccharide*.

In the group of hypertensive patients, there were no significant differences in the prevalence rates of elevated intestinal barrier markers among patients with different cardiovascular risk levels (Supplementary Table 1).

### DAO is a Primary Marker Associated With Hypertension-Related Factors

Based on the evidence of barrier markers in hypertensive patients, logistic regression analyses were performed to test the association between barrier impairment and hypertension-related factors. We compared this with patients with a hypertension history of fewer than 20 years, the prevalence of elevated DAO but not LPS, and _D_-lactate in patients with long-term hypertension (≥20 years) (53.85 vs. 23.19%, *P* = 0.040, Table 3).

**Table 3:** Intestinal barrier markers in patients with different durations of hypertension.

	**Duration ≥20 years**	**Duration <20 years**	***P*-value**
	**(*n* = 13)**	**(*n* = 69)**	
Elevated DAO	7 (53.85)	16 (23.19)	0.040
Elevated LPS	3 (23.08)	17 (24.64)	1.000
Elevated _D_-lactate	9 (69.23)	48 (69.57)	1.000

*Data are presented as n (%). DAO, diamine oxidase; LPS, lipopolysaccharide*.

After adjustment, logistic regression analysis showed that hypertension history ≥20 years (OR = 5.92, *P* = 0.028), poor DBP control (OR = 8.93, *P* = 0.011), cardiac complication (OR = 12.00, *P* = 0.024), renal complication (OR = 8.01, *P* = 0.034), and use of multiple antihypertensive medication (OR = 3.61, *P* = 0.010) were risk factors for elevated DAO (Table 4). In addition, the logistic regression analyses showed that use of multiple antihypertensive medication was associated with elevated _D_-lactate after adjustment (OR = 3.24, *P* = 0.011) (Table 4).

**Table 4:** Logistic regression analyses of risk of impaired intestinal barrier due to hypertension-related factors.

	**OR**	**95% CI**	***P*-value**
Elevated DAO			
Duration ≥20 years	5.92	1.22–28.78	0.028
Poor DBP control	8.93	1.65–48.35	0.011
Cardiac complication	12.00	1.39–103.44	0.024
Renal complication	8.01	1.17–54.69	0.034
Use of multiple antihypertensive medications	3.61	1.36–9.58	0.010
Elevated _D_-lactate			
Use of multiple antihypertensive medications	3.24	1.31–7.98	0.011

*Adjusted for gender, age, body mass index, diabetes, liver disease, history of smoking, alcohol consumption, Helicobacter pylori infection, cardiovascular risk, hypertension duration, blood pressure control, hypertensive complications, and use of multiple hypertension medications. CI, confidence interval; DAO, diamine oxidase; DBP, diastolic blood pressure; LPS, lipopolysaccharide; OR, odds ratio*.

### Association Between Blood Pressure Control and Hypertensive-Related Complication With Intestinal Barrier Impairment in Hypertensive Patients

The association between blood pressure control and the intestinal barrier biomarker was further determined. Compared with patients with good DBP control, those with poor control showed a trend toward a higher prevalence of elevated DAO, although there is no significant difference (43.48 vs. 24.10%, *P* = 0.068, Table 5). In addition, no significant relationship was found between DBP or SBP control and the other intestinal barrier markers (Table 5).

**Table 5:** Intestinal barrier functions in patients with good or poor control of blood pressure.

	**Good SBP control**	**Poor SBP control**	***P*-value**	**Good DBP control**	**Poor DBP control**	***P*-value**
	**(*n* = 67)**	**(*n* = 39)**	**(*n* = 83)**	**(*n* = 23)**	
Elevated DAO	20 (29.85)	10 (25.64)	0.643	20 (24.10)	10 (43.48)	0.068
Elevated LPS	13 (19.40)	11 (28.21)	0.296	19 (22.89)	5 (21.74)	0.907
Elevated _D_-lactate	45 (61.16)	31 (79.49)	0.174	61 (73.49)	15 (65.22)	0.436

*Data are presented as n (%). DAO, Diamine oxidase; DBP, diastolic blood pressure; LPS, lipopolysaccharide; SBP, systolic blood pressure*.

## Discussion

Our findings show that hypertensive patients had higher prevalence rates of elevated DAO and LPS than normotensive subjects, which indicates that hypertension is associated with serious intestinal barrier impairment, including small intestinal epithelium injury and endotoxin translocation. These important findings might improve early diagnosis and potential therapeutic target for hypertension-related intestinal damage in hypertensive patients. To the best of our knowledge, this is the first study to assess the association between hypertension and intestinal barrier integrity in humans.

These clinical findings (Table 2, Figure 1) are consistent with those in animal models, which showed that hypertension was associated with obvious intestinal pathology. Santisteban and colleagues tested gut epithelial integrity and gut wall pathology in SHRs (12) and found that high BP was associated with morphological changes (e.g., decreased goblet cells and shorter villi), a leaky intestinal barrier (involving increased permeability and decreased tight junction proteins), and increased small intestine inflammation. Notably, the intestinal barrier impairment in this animal model was reversed by antihypertensive medications (12). Jaworska and colleagues reported similar results using the same animal model (11). In addition, the antihypertensive drug candesartan attenuates hypertension-induced gut leakage (22).

Several mechanisms might explain why hypertensive patients have intestinal barrier impairments. First, hypertension can cause decreased intestinal blood flow (11, 12). One study showed that elevated BP in hypertensive rats could cause changes in arterioles, including thicker walls and smaller lumens, which can decrease intestinal blood flow (11). Additionally, clinical research showed that hypertension-induced vascular pathologies (such as arterial stiffness, atherosclerosis, and vascular stenosis) could reduce intestinal perfusion (19, 23). Chronic ischemia due to decreased perfusion can cause intestinal mucosa damage and barrier impairment (24). Second, gut microbiota dysbiosis is frequently found in hypertensive patients, although the causal relationship needs to be further investigated (25). Compared with normotensive subjects, hypertensive patients have dramatically decreased microbial richness and diversity, reduced short-chain fatty acid production, and overgrowth of opportunistic pathogens such as *Klebsiella* spp. and *Streptococcus* spp. (25, 26). These changes weaken the intestinal barrier by reducing short-chain fatty acid levels (which enhance intestinal barrier integrity and inhibit gut inflammation) and can even directly damage the intestinal epithelium (27, 28). Finally, hypertension-induced vascular endothelial cell injury is associated with subepithelial mononuclear cell infiltration (11). Inflammatory mediators and reactive oxygen species subsequently released by inflammatory cells can trigger intestinal barrier injury (24).

Vice versa, intestinal barrier dysfunction plays an important role in the etiology of hypertension. Gut microbiota is considered as a composition and regulator of the intestinal barrier, which plays a critical role in the metabolism and maturation of the immune system (29). A leaky intestinal barrier may promote LPS and metabolites of gut microbiota to the host, thereby inducing host inflammation and, finally, cardiovascular events (18, 30). Studies have shown that the composition and richness of gut microbiota were changed in hypertension patients, and transplantation of feces from hypertensive subjects could induce hypertension status in germ-free mice (26). In addition, antibiotics treatment contributes to dysbiosis of gut microbiota, alteration of bacteria-produced short-chain fatty acids, and an increase in the blood pressure in the experimental animal model (31). Prominently, a recent study has proven that the impairment of the intestinal barrier was due to cirrhosis and not hypertension itself. Farnesoid x receptor agonists can protect barrier function and thereby reduce gut bacterial translocation to host circulation in mice models (32). These studies provide the possibility of prevention for hypertension through a novel gastrointestinal barrier pathway. To be mentioned, the mosaic history indicates hypertension has resulted from multiple reasons, including genes, the immune system, the endocrine system, and the environment (33). The gut and gut microbiota, one of the biggest organs and “the forgotten organ” (34), might be underestimated in hypertension and hypertension-related organ damage.

The enzyme DAO is mainly expressed in intestinal mucosa and usually has a low presence in blood. The amount of DAO is positively associated with the permeability of the intestinal barrier (35). Previous study has shown increased activity of DAO in SHRs, and the increase of BP might be related to degradation of putrescine by DAO (36). Another study connected heart function with the gastrointestinal tract, showing that the plasma level of DAO was significantly elevated after cardiopulmonary resuscitation (CPR) in porcines (37). To the best of knowledge, our present study is the first study in human that shows that increased DAO in circulation is positively associated with hypertension (Table 2).

Our results show that impaired intestinal barrier is associated with multiple hypertension-related factors and complications (Table 4). Intestinal barrier injury in long-term hypertension (>20 years) might be a result of organ failure or even a direct complication of hypertension (7, 38). DBP is a risk factor for multiple cardiovascular diseases; similarly (39, 40), our results showed that higher DBP was a risk factor for small intestinal mucosal injury. Moreover, poor DBP control may indicate that patients might have increased vascular stiffness or arteriosclerosis, which can decrease intestinal perfusion; interestingly, the lack of perfusion after CPR may induce a decrease of DAO and impaired intestinal integrity (37). The present study also suggests that hypertension-related renal and cardiac complications are risk factors for intestinal mucosa impairment. Accumulation of toxins in the blood and urea in the intestine disrupts intestinal tight junction proteins, decreases epithelial barrier function, impairs gut homeostasis, and triggers intestinal disorders in chronic kidney diseases (7, 41). In addition, heart failure leads to severe congestion and disordered microcirculation in the gut (6). These hemodynamic alterations lead to intestinal epithelial edema and dysfunction due to increased hypoxia (42).

In conclusion, the present study indicates that hypertension is associated with biomarkers of intestinal barrier impairment, especially in patients with long hypertension duration, poor BP control, cardiac and renal complications, and the use of multiple antihypertensive medications. Further studies are required to determine the precise mechanism of the intestinal barrier, serum biomarkers, and the gut microbiome in hypertension and, finally, to gain insights into diagnosis or therapy for hypertensive patients.

There are several limitations to the present study. First, the results were generated from a single-center study. Second, due to the retrospective nature of our study, some confirmatory examinations, such as endoscopic and mucosal pathologic examinations, were not available for most patients. Serum markers only indirectly reflect intestinal barrier function. Finally, more cases of participants as well as mechanism studies are required to generate a confirmatory conclusion.

## Data Availability Statement

All datasets generated for this study are included in the article/Supplementary Material.

## Ethics Statement

This study was performed in accordance with the Declaration of Helsinki and with the approval of the Ethical Committee of the First Affiliated Hospital of Guangdong Pharmaceutical University. Given that this was a retrospective study, informed consent from the research subjects was waived.

## Author Contributions

CL, DL, and PX designed the study and carried out the data collection. H-JZ and X-XH designed the study. All authors wrote the paper, read, and approved the final manuscript.

## Conflict of Interest

The authors declare that the research was conducted in the absence of any commercial or financial relationships that could be construed as a potential conflict of interest.

## Abbreviations

BMI: body mass index
BP: blood pressure
CI: confidence intervals
DAO: diamine oxidase
DBP: diastolic blood pressure
LPS: lipopolysaccharide
OR: Odds ratio
SBP: systolic blood pressure
SHR: spontaneously hypertensive rat.

## References

[1] Lu J Lu Y Wang X Li X Linderman GC Wu C Prevalence, awareness, treatment, and control of hypertension in China: data from 1.7 million adults in a population-based screening study (China PEACE Million Persons Project). Lancet. (2017) 390:2549–58. 10.1016/S0140-6736(17)32478-9 29102084

[2] Whelton PK Carey RM Aronow WS Casey DE Jr Collins KJ Dennison Himmelfarb C 2017 ACC/AHA/AAPA/ABC/ACPM/AGS/APhA/ASH/ASPC/NMA/PCNA guideline for the prevention, detection, evaluation, and management of high blood pressure in adults: executive summary: a report of the American College of Cardiology/American Heart Association Task Force on Clinical Practice Guidelines. Circulation. (2018) 138:e426–83. 10.1161/CIR.0000000000000597 30354655

[3] Collaboration NCDRF Worldwide trends in blood pressure from 1975 to 2015: a pooled analysis of 1479 population-based measurement studies with 19.1 million participants. Lancet. (2017) 389:37–55. 10.1016/S0140-6736(16)31919-5 27863813 PMC5220163

[4] Kelly TN Bazzano LA Ajami NJ He H Zhao J Petrosino JF. Gut microbiome associates with lifetime cardiovascular disease risk profile among Bogalusa Heart Study participants. Circ Res. (2016) 119:956–64. 10.1161/CIRCRESAHA.116.309219 27507222 PMC5045790

[5] Tang WH Kitai T Hazen SL. Gut microbiota in cardiovascular health and disease. Circ Res. (2017) 120:1183–96. 10.1161/CIRCRESAHA.117.309715 28360349 PMC5390330

[6] Kamo T Akazawa H Suzuki JI Komuro I. Novel concept of a heart-gut axis in the pathophysiology of heart failure. Korean Circ J. (2017) 47:663–9. 10.4070/kcj.2017.0028 28955383 PMC5614941

[7] Yang T Richards EM Pepine CJ Raizada MK. The gut microbiota and the brain-gut-kidney axis in hypertension and chronic kidney disease. Nat Rev Nephrol. (2018) 14:442–56. 10.1038/s41581-018-0018-2 29760448 PMC6385605

[8] Yu LC. Microbiota dysbiosis and barrier dysfunction in inflammatory bowel disease and colorectal cancers: exploring a common ground hypothesis. J Biomed Sci. (2018) 25:79. 10.1186/s12929-018-0483-8 30413188 PMC6234774

[9] Drolia R Bhunia AK Crossing the intestinal barrier *via* listeria adhesion protein and internalin A. Trends Microbiol. (2019) 27408–25. 10.1016/j.tim.2018.12.007 30661918

[10] Tilg H Zmora N Adolph TE Elinav E. The intestinal microbiota fuelling metabolic inflammation. Nat Rev Immunol. (2020) 20:40–54. 10.1038/s41577-019-0198-4 31388093

[11] Jaworska K Huc T Samborowska E Dobrowolski L Bielinska K Gawlak M. Hypertension in rats is associated with an increased permeability of the colon to TMA, a gut bacteria metabolite. PLoS ONE. (2017) 12:e0189310. 10.1371/journal.pone.0189310 29236735 PMC5728578

[12] Santisteban MM Qi Y Zubcevic J Kim S Yang T Shenoy V. Hypertension-linked pathophysiological alterations in the gut. Circ Res. (2017) 120:312–23. 10.1161/CIRCRESAHA.116.309006 27799253 PMC5250568

[13] Jin X Yu CH Lv GC Li YM. Increased intestinal permeability in pathogenesis and progress of nonalcoholic steatohepatitis in rats. World J Gastroenterol. (2007) 13:1732–6. 10.3748/wjg.v13.i11.1732 17461479 PMC4146955

[14] Rainer F Horvath A Sandahl TD Lebe B Schmerboeck B Blesl A. Soluble CD163 and soluble mannose receptor predict survival and decompensation in patients with liver cirrhosis, and correlate with gut permeability and bacterial translocation. Aliment Pharmacol Ther. (2018) 47:657–64. 10.1111/apt.14474 29266346 PMC6333289

[15] Kim S Goel R Kumar A Qi Y Lobaton G Hosaka K. Imbalance of gut microbiome and intestinal epithelial barrier dysfunction in patients with high blood pressure. Clin Sci. (2018) 132:701–18. 10.1042/CS20180087 29507058 PMC5955695

[16] Tulkens J Vergauwen G Van Deun J Geeurickx E Dhondt B Lippens L. Increased levels of systemic LPS-positive bacterial extracellular vesicles in patients with intestinal barrier dysfunction. Gut. (2020) 69191–3. 10.1136/gutjnl-2018-317726 30518529 PMC6943244

[17] Peng JH Leng J Tian HJ Yang T Fang Y Feng Q. Geniposide and chlorogenic acid combination ameliorates non-alcoholic steatohepatitis involving the protection on the gut barrier function in mouse induced by high-fat diet. Front Pharmacol. (2018) 9:1399. 10.3389/fphar.2018.01399 30618733 PMC6298419

[18] Zhou X Li J Guo J Geng B Ji W Zhao Q. Gut-dependent microbial translocation induces inflammation and cardiovascular events after ST-elevation myocardial infarction. Microbiome. (2018) 6:66. 10.1186/s40168-018-0441-4 29615110 PMC5883284

[19] Williams B Mancia G Spiering W Agabiti Rosei E Azizi M Burnier M 2018 ESC/ESH guidelines for the management of arterial hypertension: the task force for the management of arterial hypertension of the European Society of Cardiology and the European Society of Hypertension: the task force for the management of arterial hypertension of the European Society of Cardiology and the European Society of Hypertension. J Hypertens. (2018) 36:1953–2041. 10.1097/HJH.0000000000001940 30234752

[20] Schmieder RE. End organ damage in hypertension. Dtsch Arztebl Int. (2010) 107:866–73. 10.3238/arztebl.2010.0866 21191547 PMC3011179

[21] Fenzl A Itariu BK Kosi L Fritzer-Szekeres M Kautzky-Willer A Stulnig TM Circulating betatrophin correlates with atherogenic lipid profiles but not with glucose and insulin levels in insulin-resistant individuals. Diabetologia. (2014) 571204–08. 10.1007/s00125-014-3208-x 24623100

[22] Wu D Tang X Ding L Cui J Wang P Du X. Candesartan attenuates hypertension-associated pathophysiological alterations in the gut. Biomed Pharmacother. (2019) 116:109040. 10.1016/j.biopha.2019.109040 31170664

[23] Nakanishi R Baskaran L Gransar H Budoff MJ Achenbach S Al-Mallah M. Relationship of hypertension to coronary atherosclerosis and cardiac events in patients with coronary computed tomographic angiography. Hypertension. (2017) 70:293–9. 10.1161/HYPERTENSIONAHA.117.09402 28607128 PMC5518701

[24] Granger DN Holm L Kvietys P The gastrointestinal circulation: physiology and pathophysiology. Compr Physiol. (2015) 51541–83. 10.1002/cphy.c150007 26140727

[25] Yan Q Gu Y Li X Yang W Jia L Chen C Alterations of the gut microbiome in hypertension. Front Cell Infect Microbiol. (2017) 7:381 10.3389/fcimb.2017.00381 28884091 PMC5573791

[26] Li J Zhao F Wang Y Chen J Tao J Tian G Gut microbiota dysbiosis contributes to the development of hypertension. Microbiome. (2017) 514 10.1186/s40168-016-0222-x PMC5286796 28143587

[27] Geirnaert A Calatayud M Grootaert C Laukens D Devriese S Smagghe G. Butyrate-producing bacteria supplemented in vitro to Crohn's disease patient microbiota increased butyrate production and enhanced intestinal epithelial barrier integrity. Sci Rep. (2017) 7:11450. 10.1038/s41598-017-11734-8 28904372 PMC5597586

[28] Potgens SA Brossel H Sboarina M Catry E Cani PD Neyrinck AM. Klebsiella oxytoca expands in cancer cachexia and acts as a gut pathobiont contributing to intestinal dysfunction. Sci Rep. (2018) 8:12321. 10.1038/s41598-018-30569-5 30120320 PMC6098145

[29] Takiishi T Fenero CIM Camara NOS. Intestinal barrier and gut microbiota: shaping our immune responses throughout life. Tissue Barriers. (2017) 5:e1373208. 10.1080/21688370.2017.1373208 28956703 PMC5788425

[30] Anker SD Egerer KR Volk HD Kox WJ Poole-Wilson PA Coats AJ. Elevated soluble CD14 receptors and altered cytokines in chronic heart failure. Am J Cardiol. (1997) 79:1426–30. 10.1016/S0002-9149(97)00159-8 9165177

[31] Pluznick JL Protzko RJ Gevorgyan H Peterlin Z Sipos A Han J. Olfactory receptor responding to gut microbiota-derived signals plays a role in renin secretion and blood pressure regulation. Proc Natl Acad Sci USA. (2013) 110:4410–5. 10.1073/pnas.1215927110 23401498 PMC3600440

[32] Sorribas M Jakob MO Yilmaz B Li H Stutz D Noser Y. FXR modulates the gut-vascular barrier by regulating the entry sites for bacterial translocation in experimental cirrhosis. J Hepatol. (2019) 71:1126–40. 10.1016/j.jhep.2019.06.017 31295531

[33] Page IH. The mosaic theory of arterial hypertension–its interpretation. Perspect Biol Med. (1967) 10325–33. 10.1353/pbm.1967.0031 6034593

[34] O'Hara AM Shanahan F. The gut flora as a forgotten organ. EMBO Rep. (2006) 7688–93. 10.1038/sj.embor.7400731 16819463 PMC1500832

[35] Wolvekamp MC de Bruin RW. Diamine oxidase: an overview of historical, biochemical and functional aspects. Dig Dis. (1994) 122–14. 10.1159/000171432 8200121

[36] Perin A Sessa A Desiderio MA. Polyamine levels and diamine oxidase activity in hypertrophic heart of spontaneously hypertensive rats and of rats treated with isoproterenol. Biochim Biophys Acta. (1983) 755:344–51. 10.1016/0304-4165(83)90236-2 6218830

[37] Lu Y Li CS Wang S. Effect of hypertransfusion on the gastrointestinal tract after cardiac arrest in a porcine model. World J Emerg Med. (2012) 349–54. 10.5847/wjem.j.issn.1920-8642.2012.01.009 25215039 PMC4129819

[38] Meijers B Jouret F Evenepoel P. Linking gut microbiota to cardiovascular disease and hypertension: lessons from chronic kidney disease. Pharmacol Res. (2018) 133:101–7. 10.1016/j.phrs.2018.04.023 29715498

[39] Sundstrom J Neovius M Tynelius P Rasmussen F. Association of blood pressure in late adolescence with subsequent mortality: cohort study of Swedish male conscripts. BMJ. (2011) 342:d643. 10.1136/bmj.d643 21343202 PMC3042737

[40] de Waard DD de Borst GJ Bulbulia R Huibers A Halliday A Asymptomatic Carotid Surgery Trial-1 Collaborative G. Diastolic blood pressure is a risk factor for peri-procedural stroke following carotid endarterectomy in asymptomatic patients. Eur J Vasc Endovasc Surg. (2017) 53:626–31. 10.1016/j.ejvs.2017.02.004 28318997 PMC5423873

[41] Vaziri ND Yuan J Norris K. Role of urea in intestinal barrier dysfunction and disruption of epithelial tight junction in chronic kidney disease. Am J Nephrol. (2013) 371–6. 10.1159/000345969 23258127 PMC3686571

[42] Sandek A Bauditz J Swidsinski A Buhner S Weber-Eibel J von Haehling S. Altered intestinal function in patients with chronic heart failure. J Am Coll Cardiol. (2007) 50:1561–9. 10.1016/j.jacc.2007.07.016 17936155

