# Supplementary material for: Risk Factors for Intestinal Barrier Impairment in Patients With Essential Hypertension
Source: Front Med (Lausanne). 2021 Jan 27;7:543698. doi: 10.3389/fmed.2020.543698 (PMC7873557; doi:10.3389/fmed.2020.543698)
Supplement: Supplementary file 1 [file Table_1.docx]

**Supplementary Table 1.** Intestinal barrier functions in patients among different cardiovascular risk groups.

|  | Low risk  (n = 6) | Medium risk  (n = 8) | High risk  (n = 21) | Very high risk  (n = 71) | *P*-value |
| --- | --- | --- | --- | --- | --- |
| Elevated DAO | 2 (33.33) | 3 (37.50) | 8 (38.10) | 17 (23.94) | 0.559 |
| Elevated LPS | 0 (0) | 4 (50.00) | 3 (14.29) | 17 (23.94) | 0.108 |
| Elevated _D_-lactate | 3 (50.00) | 7 (87.50) | 14 (66.67) | 52 (73.24) | 0.437 |

Data are presented as n (%). DAO, diamine oxidase; LPS, lipopolysaccharide.
